# An artificial neural network explains how bats might use vision for navigation

**DOI:** 10.1038/s42003-022-04260-5

**Published:** 2022-12-03

**Authors:** Aya Goldshtein, Shimon Akrish, Raja Giryes, Yossi Yovel

**Affiliations:** 1 School of Zoology, Faculty of Life Sciences, Tel Aviv University, Tel Aviv, 6997801 Israel; 2 School of Electrical Engineering, Faculty of Life Sciences, Tel Aviv University, Tel Aviv, 6997801 Israel; 3 Sagol School of Neuroscience, Tel Aviv University, Tel Aviv, 6997801 Israel; 4 Present Address: Department of Collective Behavior, Max Planck Institute of Animal Behavior, Konstanz, 78464 Germany

**Keywords:** Animal behaviour, Sensorimotor processing

## Abstract

Animals navigate using various sensory information to guide their movement. Miniature tracking devices now allow documenting animals’ routes with high accuracy. Despite this detailed description of animal movement, how animals translate sensory information to movement is poorly understood. Recent machine learning advances now allow addressing this question with unprecedented statistical learning tools. We harnessed this power to address visual-based navigation in fruit bats. We used machine learning and trained a convolutional neural network to navigate along a bat’s route using visual information that would have been available to the real bat, which we collected using a drone. We show that a simple feed-forward network can learn to guide the agent towards a goal based on sensory input, and can generalize its learning both in time and in space. Our analysis suggests how animals could potentially use visual input for navigation and which features might be useful for this purpose.

## Abstract (web summary)

A machine learning model is trained to navigate a visual environment in a similar way to a bat, demonstrating similarities between trained computer models and the mammalian brain.

## Introduction

With over a century of navigation research, we now have a better understanding of how accurate navigation must be to support complex movement such as daily commute flights and migration^1–3^. On the one hand, we know much about which sensory modalities animals use for navigation^4–7^. But on the other hand, how animals use this sensory information in order to navigate, that is, how animals translate sensory input into movement, is poorly understood. A few previous attempts tried to examine this question theoretically, developing models that explain how an animal can use the sensory information that is available to it in order to navigate^8–12^. Yet, we are still very far from answering this question. In this study, we examined the problem from the point of view of visual navigators^13,14^—Egyptian Fruit bats (*Rousettus aegyptiacus*), hereinafter referred to as the “bats”. These bats perform nightly long-range commutes up to several dozens of kilometers from their roost, navigating toward specific fruit trees in a remarkably direct trajectory while probably relying on vision^13,14^. Importantly, the bats cannot see their target, that is, a specific fruit tree (or sense it using other sensory systems) until they are very close to it, so they must somehow acquire and use other visual information which is available along the way in order to navigate toward the tree. Although theoretically these bats can use other sensory modalities to navigate, such as echolocation, olfaction or magnetic sensing, it is unlikely that they did so during this type of navigation task for the following reasons: (1) They commuted in high altitudes above the ground (>100 m) usually without emitting echolocation calls^15,16^, probably because ground (or other objects) cannot be detected at this range^17^. (2) It is improbable that the bats can smell a specific tree (one of many thousands of the same species) from 20 km. Moreover, relying on olfactory gradients (e.g., the ocean might provide such a gradient) is expected to lead to less direct navigation than the one we observed. Notably, fruit bats might use olfaction to home-in on a specific fruit during the final few meters of its flight, but this is not what we modeled in this study. (3) Magnetic sensing could be used as a compass during navigation^18–20^, but probably not as a map. In any case, the purpose of this work was to test visual-based navigation which is a known strategy used by these bats^13,14^.

We used a machine learning approach in order to study if and how visual information could be used by an animal to learn to navigate along a route to a familiar goal. Machine learning algorithms are very powerful for statistical learning, providing new opportunities for studying animal behavior. Moreover, several studies suggested that the architecture of convolutional neural networks somewhat resembles mammalian visual processing^21–23^. The network is composed of several layers of convolution filters which extract feature from the visual scene, similar to the mammalian visual cortex. On the other hand, the network only performs feedforward computation, thus making it less powerful in comparison to the recurrent mammalian brain^24–27^. We refer to the units in the network as neurons and analyze their encoding of space. A handful of previous studies used machine learning algorithms to study animal navigation, e.g., in ants, honeybees, and rats^12,24–27^, mostly focusing on short-range navigation in the order of dozens of meters^24^ (which sometimes might be considered long-range navigation for the animal conducting the task). Herein this study, we use artificial neural networks to study visual navigation over a larger scale of dozens of kilometers using the actual visual information available to a fruit bat in the wild.

**Fig. 1:**
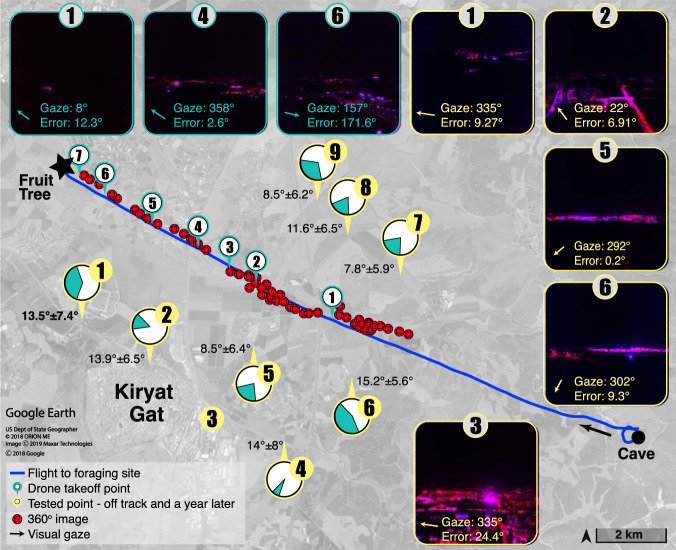
Navigation-net can explain visual animal navigation. The blue line shows the bat’s actual trajectory from its cave (black circle) to its target tree (black star). Red spheres show points where the drone collected 360° images (the drone took off at green points 1–7). Images with green frames show three examples of input-images with the actual (allocentric) direction in which they were taken, i.e., the gaze relative to the target (solid arrow) and the output error in degrees relative to the target. The error is depicted in the image. Gaze angles are given using a 0–360° clockwise notation with 0° pointing toward the fruit tree target. The figure also shows the locations where 360° images were taken a year later to test generalization (yellow points 1–9) with the region where the network performed well shown in green and the error in degrees given for these green sectors. Five example input images taken in the yellow points are shown on the right side (yellow frame) with the gaze and error angles depicted on them too. Note that in point 3 the navigation-net was not able to point toward the direction of the target with an error of less than 24° at all. The satellite image was obtained from Google Earth, 2018.

To acquire the visual information available to a navigating bat, we flew a drone along the exact path flown by a specific individual bat, at a height of 100 m above the ground, which is typical for these bats (“Methods”). While flying the drone, we recorded the 360 degrees (panoramic) visual information that the bat could rely on for navigation (Fig. 1 and “Methods”). We then degraded the visual information, making sure that we underestimated what was available for a bat. We next trained a very simple convolutional neural network (CNN) to navigate this route (Supplementary Table 1). The network receives the visual input at a specific location and heading along the route, and outputs the azimuth of the goal (which cannot be seen in the image), that is, how much should the bat turn in order to head toward its target-fruit tree.

We show that the network can learn to navigate along a typical bat’s route, even though the visual information dramatically changes along the way, for example, the light conditions and landscape complexity change (e.g., switch from rural to city backgrounds) and the salient available information changes from distal to proximal cues. We also show that the network can generalize both in space and in time, thus suggesting how a bat could deal with wandering several kilometers to an unknown location off-track or with returning to use the same path after not using it for many months. We also discuss which visual features might be used by a navigating bat, and we analyze the units in the trained neural network in comparison to what is known about spatial encoding in the mammalian brain.

## Results

### Navigation-net explains visual navigation

We trained a convolutional neural network (CNN, henceforth the navigation-net) to reproduce bat navigation based on visual information. The network received a single image taken anywhere in any direction along the bat’s route as input, and it was trained to provide the angle to the target-foraging tree as output. This output angle represents the angular error between the current gaze of the bat (the angle at which the image was taken) and the azimuth of the bat’s desired target (Fig. 1). Note that for an aerial navigator that mostly can freely change its direction, such as a bat, the angle to the target is the desired information for navigation, while terrestrial navigators are sometimes required to conduct detours around obstacles. We made sure that the network based its decision on visual data that is available to a bat. To this end: (1) the network received as input a single image covering a field of view of 80° to output direction (narrower than the field of view of a bat^28^, and note that the real bats can turn its head and integrate information before making guidance decisions); (2) image resolution was reduced to 160 × 160 pixels (less than that available for a bat^28^), and (3) we only used the red and blue channels of the images in accordance with the photoreceptors in the retina of these bats^29^, but see “Methods” for more details.

We first tested the navigation-net’s performance when trained with data along the entire trajectory (i.e., both training and testing images were taken from the same set). This scenario, which mixes training and testing data, might be considered as overfitting the network by the machine learning community. However, in our case, we examined whether a bat can learn to navigate a specific trajectory based on the available visual input, as fruit bats typically do when flying repeatedly to the same goal along the same route for dozens of nights. We were thus not concerned about overfitting the network at this stage of the study. Below, we also report testing the network’s ability to generalize its learning.

### Navigation-net performance

**Fig. 2:**
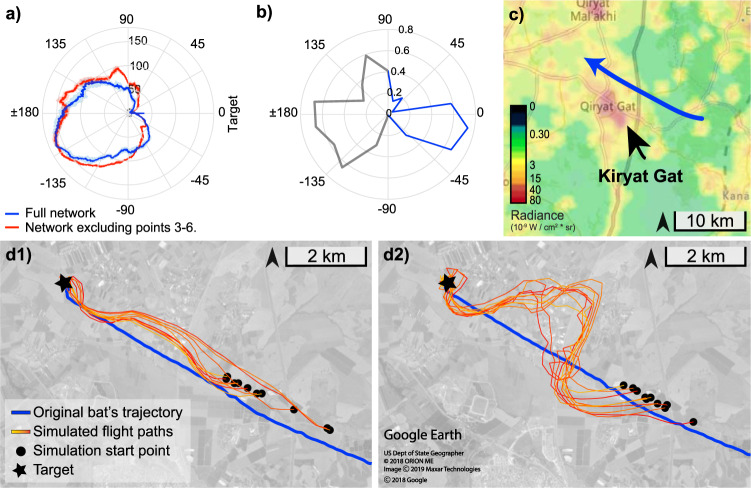
Navigation performance. **a** The performance of the network as a function of the angle relative to the target is plotted as a polar plot. Data were collected from seven points along the bat’s trajectory. Error represents the mean error over all test images taken randomly along the route. 2521 images for the blue line depicting the error of the network trained on all data; and 2517 images for the red line depicting the error of the network trained on all data excluding points 3–6. Positive angles represent angles between 0 and 180° and negative angles are between 180 and 360°. **b** Percentage of light at a certain angle, relative to the entire measured area, in a distance of up to 15 km from the cave (illumination levels were measured in units of radiance 10^−9^ W/cm^2^ × sr. 0 degrees represent the direction of the target relative to the cave). Note that bats probably rely on visual information in the direction of their flight (blue line) and thus do not rely on visual information that is greater than ±90° of their flight direction (gray line). **c** The bat’s trajectory (blue line) is overlaid on the background of an artificial lightmap. The lightmap was obtained from Jurij Stare, using NASA’s Black Marble nighttime lights product. **d** Trajectories of the bat-simulator show network-based navigation for the network that was trained on all data (**d1**) and on part of the data (**d2**). Ten examples are shown for each network. Note how the network trained with partial data (**d2**) has a much larger error between points 3 and 6, a route that the network has not ‘seen’ before, but it eventually manages to reach the target (black star). The satellite images were obtained from Google Earth, 2018.

The network was able to learn the task with high accuracy—the mean error over a sector of ±45° relative to the target was 22.2° ± 19.7° (mean ± SD, *n* = 606, Fig. 1 and Fig. 2a, blue line). This shows that a single network can be trained to memorize a specific navigation trajectory based on relevant visual input, even though the visual input dramatically changes along the path (note how different images 1, 4 in Fig. 1 are, even though they were taken at similar gaze angles relative to the target; see more examples in Supplementary Fig. 1). The network was most accurate when receiving input images from small gaze angles, that is, when looking at the direction of the target (Fig. 2a). This is probably a result of the fact that our training set was biased, with more images taken in the direction of the target—we rotated the drone in all directions, but when it ascended or moved north/south to the acquisition point, it was always roughly facing the goal’s direction. This is probably also the case with a real bat that flies toward a specific target for many nights and faces the target most of the time, thus likely learns the trajectory according to information in the direction of the target while gathering less accurate information areas about other angles. We moreover found that the network’s error was not symmetric around the azimuth of the target and was slightly higher for negative angles (leaning toward the south). This might be a result of the extent light sources in that direction, as can be learned from analyzing the light-map of the region (Fig. 2b, c).

We have shown that the network can memorize a specific trajectory, but there are many scenarios in which memorizing highly specific sensory input might be detrimental for navigation: (1) Animals sometimes navigate along a route not traveled for a long time, during which sensory input might change. This is especially relevant for migrating species that navigate along a specific route yearly but also for many foragers that rely on seasonally changing resources such as fruit trees that are ripe once a year. (2) Animals might sometimes also find themselves off-route needing to return to it, for example, when drifting due to side winds or when intentionally wandering in aim to explore. Thus, animals must have some ability to adjust to changing sensory input and to generalize their navigation in light of changing sensory input. To test navigation-net’s generalization abilities, we flew the drone one year later than the original acquisition at 9 locations 2–5 km away from the original trajectory, acquiring images from 360 degrees at each point (yellow points in Fig. 1), and tested its ability to find the target based on training on images taken one year earlier. This test thus examined generalization both in space and in time. Notably, visual input changed much throughout this year, with numerous new buildings built in the region. In each of the tested locations (except for the one in the center of a city, see point 3 in Fig. 1), the network was able to point toward the direction of the target with a minor error of less than 15 degrees for at least part (on average 79.4° ± 50.1°) of the field of view (see green sectors and precise errors in Fig. 1). Because a bat can turn its head while flying, this result suggests that in any of these locations, a bat could estimate the direction to the target even after not visiting the area for one year (see more in the “Discussion”).

Navigation generalization is also crucial when learning a new route for the first time. Egyptian fruit bats typically explore extensively in search of new foraging trees. These long exploration flights are characterized by high tortuosity, while later on, when the animal returns to a specific tree that it found on a previous night, it will do so in a much more direct trajectory (see Fig. 1, panel d in ref. ^13^). Thus, the animal shows an ability to navigate directly along a route it has never seen before. We tested navigating-net’s ability to replicate this behavior by training a new network using the same approach described above, but this time excluding part of the data from training. Specifically, when training the new network, we excluded all visual input acquired along the ~4 km between points 3 and 6 (~23% of the entire trajectory, see Fig. 1). We then tested the network’s performance only on images taken between points 4 and 5, thus testing the network on images that were at least 1 km away from any image it has seen before (Supplementary Fig. 1).

The network was able to perform the task with high accuracy even when tested on images it was never trained with—the mean error over a sector of ±45° relative to the target was 25.4° ± 19.3° (*n* = 592 test images, see red line in Fig. 2a). This error is larger than that achieved for the previously trained network, but it should be sufficient for navigating to the target, as we also demonstrate in the next paragraph.

### The bat-simulator

So far, we tested navigation-net’s performance at single view-points. To examine whether this performance is sufficient for real-world multi-stage navigation, we programmed a bat-simulator that was based on navigation-net. In this simulation, the bat was placed at a random position within the first 2.5 km of the trajectory with a random heading drawn from an even distribution of [−30°, 30°] relative to the target. At every step, the simulation used the visual input according to the location and heading of the simulated bat to determine its azimuth relative to the target using a navigation-network (see “Methods”). The simulator then turned toward this estimated azimuth, moved a distance of 350 m, and once again estimated the direction of the target using the updated visual input at the new location. We considered a simulated-flight as successful if the simulated bat started flying around the target in an ellipsoid manner. We tested both of the networks described above, one trained on all data and the other excluding the data between points 3 and 6 (50 runs for each network). In all 100 runs, the simulated bat always managed to navigate to the target (see example trajectories in Fig. 2d). Interestingly, the simulated bat trained with partial data was much less accurate, but this was at least partially related to our very simple control algorithm. A better control algorithm that takes the derivative of the movement into account^30^ will provide a smoother trajectory, but even our current simple algorithm was sufficient for navigating to the target, suggesting how animals which rely on distal sensory input can navigate along routes they have never taken before (Fig. 2d).

Furthermore, to examine which part of the images was most important for navigation, we trained the network with images in which we horizontally removed one-thirds of the images (Supplementary Fig. 2). As expected, the network performed worst when the central stripe of the images was removed. This part of the images usually contained horizon information, while the other two contained proximal ground or distal sky information. The performance for the removal of the top, middle and bottom stripes, respectively, was: 26.5° ± 26.2°, 101.5° ± 43.03°, and 16.8° ± 65.2° (for a sector of 45° relative to the target), showing that removing the top or bottom hardly influenced performance while removing the center dramatically degraded the performance.

### Navigation-net is facilitated by goal-direction neurons

**Fig. 3:**
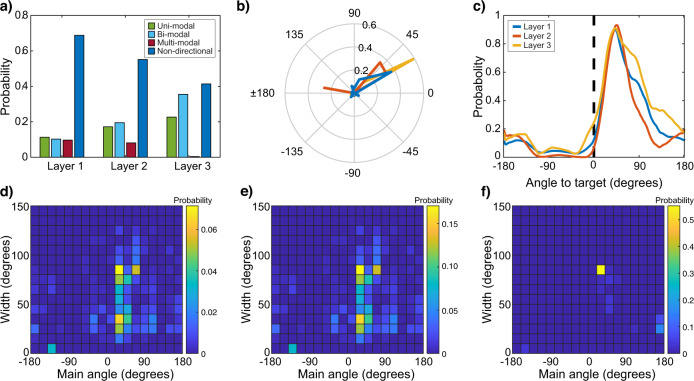
Goal-direction neurons in navigation-net. **a** Neuron types in the three last layers of navigation-net (layers 1 to 3 have 4096, 1024, and 256 neurons, respectively). **b** The distribution of the peaks of the azimuth-dependent activation patterns of unimodal neurons in the last three layers (layers 1 to 3 in blue, red, and yellow lines, respectively). **c** The average of the azimuth-dependent activation distribution of unimodal units with peaks in the angle range with the highest probability (between 20 and 40 degrees in layers 1 and 3, and between 40 and 60 degrees in layers 2). This can be thought of as the average tuning curve of most of the unimodal neurons. **d**–**f** The 2D distribution of peaks and tuning widths of unimodal neurons in the first (**d**), second (**e**), and third (**f**) layers of the network.

Finally, to gain insight about the navigation strategy learned by navigation-net, we analyzed the three last (fully-connected) layers of the neural network (Supplementary Table 1). To this end, we analyzed the azimuth-dependent activation patterns of the units in these three layers. Many neurons in these layers were characterized by unimodal, bimodal, or multimodal activation distributions, that is, neurons that encode a certain azimuth relative to the target, similar to goal-cells which were found in the mammalian brain^31^. The downstream layers in the network (layers 2–3) had more unimodal and bimodal neurons on account of neurons with multimodal and non-directional distributions (Fig. 3a). Further examination of the unimodal neurons’ distribution revealed that the peak of the activation in most of these neurons occurred for azimuths between 20° and 60° (northwest) relative to the target in all three layers (Fig. 3b, c). Notably, this is also where the error of the network was minimal (see Fig. 2a), and is probably a result of our biased acquisition approach that acquired more information in the direction of the target. The distribution of the (two-sided 6 dB) width of the neurons’ azimuth-dependent activation became wider (less tuned) in downstream layers, with wide (90°) and narrow (30°−60°) neurons at the first and second layers and only wide (90°) neurons at the last layer (Fig. 3d–f).

## Discussion

Navigation-net demonstrates how visual input available to an animal could potentially be used to guide the learning of navigation and later on to guide actual movement along a learned route to a familiar target (when only visual input is considered). The network was able to learn a specific bat navigation route based on the available visual input. This is not trivial as the visual sensory input dramatically changes along the route, and therefore, the network must sometimes output the exact same angles for completely different images (e.g., sometimes the horizon was completely occluded by the landscape, other-times a nearby village produced very bright lights). Our network’s performance cannot be explained in term of simple phototaxis as a simple strategy such as always moving toward the brightest direction would never lead to the target (this can be learned from the lightmap, Fig. 2c). The network also exhibited impressive generalization abilities both in space and in time, suggesting how animals might be able to navigate despite environmental changes in sensory input and how they can navigate along part of a route never used before, by relying on a distal familiar sensory input. We made sure that we underestimated the visual input that a real bat could use. Our network was only trained on a single trajectory, while real bats probably have to remember dozens (or more) trajectories. Notably, artificial neural networks are renowned for their ability to memorize numerous examples^32^, as was also demonstrated to some extent in our network—the network learned thousands of images and had to learn to provide the same output for completely different input images (see Supplementary Fig. 1). It is thus likely that the same network could memorize many more trajectories, but we did not test this due to the difficulty of acquiring the data. Note that training to navigate on multiple routes in the same area (e.g., the animal’s home range) would improve the animal’s (and the network’s) ability to generalize and move in new paths.

While real bats might also use alternative non-visual sensory information (e.g., olfaction) for navigation, here, we focused on visual-based navigation that is likely dominant for navigation in this species^13,14^. Moreover, a real bat might roughly memorize multiple routes and then tune its navigation memory seasonally when returning to use a path not used for a while.

In Supplementary Fig. 3, we show an example of a real bat moving toward a target not visited for a while, demonstrating how navigation improves over consecutive nights. Interestingly, this improvement is reminiscent of the ability of our bat-simulator to move along a route not taken before. That is, if we used the data acquired by the simulator during its first non-direct movements along a new route (Fig. 2d), to better train the network, it would probably have improved its navigation over consecutive nights and navigated more directly on following nights, just like the real bat in Supplementary Fig. 3 does. This behavior suggests how learning the association between the direction of a remote target and distal landmarks could work. We hypothesize that this could happen in steps through learning intermediate routes to closer targets. Indeed, behavioral evidence from our previous studies suggest that young bats gradually increase their exploration area, and use shortcuts to fly between their target fruit trees^13,33^.

In this study, we only trained the network to output the direction of the target, but it is likely that it could have also learned to output the distance to the target. Moreover, our bat-simulator suggests how navigation could be performed even without an exact estimate of the distance—the simulator only used the azimuth output of the network and managed to reach the target.

Our analysis shows that the center of the image, which contains most of the horizon information, is most useful for navigation. This is probably true in the specific region when flying toward the northwest as the bat did, because the horizon in this direction contains rich visual information provided by the many cities located on the coast. However, this does not mean that in other situations, bats cannot use local ground information (in the lower part of the image) or celestial cues (in the upper part of the image). It is likely that our over-degraded images eliminated celestial information that would be available to a real bat. Moreover, a real bat might switch between using these alternatives based on what is available, e.g., on a foggy night it might turn to use local visual cues on the ground, such as nearby lights or the local landscape (Fig. 2c).

The network’s ability to navigate along a route never taken before (Fig. 2d right) suggests that it relied on global visual information to navigate (e.g., lights on the horizon). Relying on such distal cues is probably more resilient to changes in information over time and space because the input is blurrier, and changes are less dramatic.

When examining the network’s performance a year later, at remote previously ‘unvisited’ locations (several kilometers off the route), it was able to detect the direction of the target but only in part of the field of view. From the bat’s point of view, this should be sufficient, as it can easily turn around (360 degrees) until finding the desired direction. Interestingly, the field of view that allowed detecting the target with high accuracy was always in the southwest relative to the bat (see green sectors in the yellow points in Fig. 1). This suggests that the network used (at least partially) information from this direction. One reasonable explanation is that the network was relying on the most salient familiar visual information that was available in these locations, which it has never been trained on. One of the most likely salient familiar visual information at these previously unvisited locations were the lights of ‘Kiryat Gat’—the largest city in the area, which was in the southwest relative to these locations (Figs. 1 and  2c). This is also supported by the fact that at the locations west of Kiryat-Gat (yellow points 1–2 in Fig. 1), the network does not rely on it anymore. But clearly, the network is more robust than relying on one visual feature, as can be learned from its ability to navigate from points where Kiryat Gat is not seen (see various image examples in Fig. 1). Moreover, when the images were taken from within Kiryat Gat (yellow point 3 in Fig. 1), the network was unable to determine the direction of the target (the error was always more than 25°), probably because it received visual information very different from anything that it has seen before. The biased error of the simulated bat (Fig. 2d right) can likely also be explained by the network’s reliance on information in the southwest direction. Note that the network chose to rely on Kiryat Gat, i.e., we did not explicitly train it to do so, showing how our approach can be used to extract informative features for navigation. Moreover, note that the network was not navigating toward Kiryat Gat, that is, even when using information from the southwest, it was pointing the navigator in the correct directions of the target (which was not at the southwest).

Notice that the network uses Kiryat Gat as a main landmark mainly when navigating in unfamiliar locations. However, when navigating in a familiar route, the error was actually slightly larger when heading south to the target (the direction of Kiryat Gat) than when heading north to the target (see Fig. 2a). We hypothesize that this is due to the excess of light in this direction (Fig. 2b, c), which makes the analysis of visual information more difficult. The tuning of the neurons (that were mostly active toward north to the target) also supports this. If this hypothesis is correct, this is an interesting case in which salient visual landmarks might be beneficial for navigation from unfamiliar locations and, at the same time, detrimental for fine navigation at familiar locations.

Neural networks are powerful statistical learning algorithms which should be used with caution when comparing their results to animal behavior. Neural networks can sometimes perform mistakes that seem ridiculous to a human^34^. Moreover, the architecture of the network that we used is obviously very different from that of the mammalian brain. For example, it is a feed-forward network without feedback. Despite these differences, neural network learning has several important characteristics that allow us to infer animal abilities: (1) They are statistical learning algorithms, and in this sense, they are probably more similar to the brain than any analytical model. (2) The network we used has roughly 10 million connections, far less than the mammalian brain regions involved in navigation learning^35^. Thus, our neural network can be thought of as an underestimate model for what the brain can do, both in terms of its learning abilities and in terms of its generalization abilities. Notably, if this simple version of a brain can learn to navigate and generalize, it is not surprising that a bat’s brain can do so too. (3) Our approach shows how a single network can be used for both analyzing visual input and guiding navigation. Indeed, several recent studies suggest that movement information is integrated in the primate visual cortex^36,37^. (4) The analysis of the units in the network revealed artificial neurons tuned to the direction of the goal reminiscent of goal-neurons found in the mammalian brain^31,38,39^. Note that the narrow activation width that appears in the first and second layers fits nicely to the common tuning width of goal-neurons and head direction cells that ranges between 40°^31^ and 30°−60°, respectively^40^, however, in bats that use head direction cells for 3D navigation, the width of the head direction cells is wider with ~150°^41^. Note, that although we would expect the neurons in the network to show some directionality, their tuning and distribution of preferred directions could be completely different. For example, all neurons could have been sharply tuned to a single specific angle.

Moreover, a similar phenomenon where more neurons are tuned in a direction important to the animal, was previously demonstrated in Barn owls’ auditory system, where more neurons are tuned toward the center of their gaze, serving as a Bayesian prior for sound localization^42^. Examining the properties of artificial neural network neurons might thus lead to predictions about biological systems.

Our findings thus contribute to the understanding of biological navigation and specifically relevant for other species that rely on vision to navigate along similar distances of familiar routes, such as pigeons^43–45^. To our best knowledge, this is the first study using the statistical power of novel machine learning algorithms in order to study mammalian navigation in their natural environment. Specifically, we focus on the fundamental task of translating visual input into movement, using limited biological-plausible visual information and a limited processing algorithm (only feed-forward), suggesting that a biological brain could facilitate this behavior. We show that a single neural network can learn to navigate like a bat even across a long trajectory where visual input is constantly changing and we suggest how noisy goal-direction neurons (similar to those found in the mammalian brain) could facilitate such navigation. In addition, we provide insight into how a trajectory not take before could be re-used for navigation, a task that is routinely performed by animals with seasonal movement patterns.

Machine learning models in general and specifically Artificial Neural Networks, allow studying behavior in ways that were previously impossible. The complex behavior that we modeled in this study, could probably not be modeled with any other (non-machine learning) model (surely no analytic model would have worked). Machine learning and specifically artificial neural networks are thus allowing us to address questions such as which behaviors can be performed with which sensory information (e.g., can vision explain the navigation we observed?) or what is the minimum amount of information and computation required to perform the behavior? Machine learning algorithms also allow revealing insight about the underlying extracted features which enables the behavior. Of course, in order to validate this insight, we would need to go back and forth between the predictions of the model and the behavior.

Future studies could also use network architectures that are more reminiscent of the mammalian brain, they could be generalized to other sensory modalities and organisms, and they could be elaborated to study complex forms of navigation such as map-based navigation. Moreover, artificial neural networks can also be used to study additional behaviors, as a few studies already did^12,24–27^. We thus anticipate a rapid increasing use of the power of machine learning to study behavior and we point to an increasing need to develop ways to carefully interpret their results.

## Methods

The study was performed according to the permit of the Institutional Animal Care and Use Committee (IACUC) of Tel Aviv University (Number: L-11-054).

### Data acquisition

We used a miniature GPS data-logger (Lucid, Israel) to obtain the flight trajectory of one adult male fruit bat (*Rousettus aegyptiacus*) that emerged from its cave in the ‘Beit Govrin’ National Park, Israel and flew a distance of 17.2 km to a tree located near ‘Nir Banim’, Israel (31°40.083′N, 34°44.485′E). The bat was caught when emerging from its cave using a mist net, and the GPS device was attached to its back using surgical cement. A telemetry unit (Biotrack, Dorset, UK) was attached to the GPS to assure its recovery after falling off the bat.

To obtain the visual information that was available to the bat using this flight, we flew the drone at an elevation of 100 m above ground, similar to the average altitude of the bat along this trajectory (119 ± 74 m mean ± SD). We used DJI Mavic Pro and DJI Phantom 4 drones (DJI Science and Technology, Guangdong, China). Due to flight restrictions in this area, we flew the drone at seven locations along the last 11 km of the bat’s route (see green points in Fig. 1). At each location, we flew the drone along a ~1 km track, recording video continuously. The drone zig-zagged around the trajectory of the bat in order to increase visual variability. Every 250 m, we stopped and rotated the drone 360° to acquire images from more angles.

We used the drones’ built-in cameras to acquire visual footage (12.4 M pixel cameras). The camera’s field of view is 80 degrees. Videos were recorded at 30 fps, but reduced to 10 fps for training the classifier. In total, this provided a dataset of 33k images. Notably, as the imaging was performed at night, images are mostly colorless, including shades of white and yellow. The original image resolution was 1280 × 720 or 3840 × 2160, but the video was subsampled to 160 × 160 using bi-cubic interpolation to make sure that we were not using more information than that available to a bat^28^. Egyptian fruit bats are known to possess red and blue photoreceptors^29^. Even if bats only rely on rod vision for such navigation tasks observing our images which only use the red and blue channels, suggests that they are nearly monochromatic, which would be the case for rod-based sensing.

### Training and testing the classifier

The basic network was trained and tested on all data. For the second approach, we removed all data between points 3 and 6 from the training set and tested the algorithm on images acquired between points 4 and 5 only. The label of each image—the angle of view of the camera relative to the target tree was estimated based on the drone’s flight-log files, which are updated at a rate of 10 times per second.

The network was trained using TensorFlow platform in Python^46^. The output of the navigation-net was an angle between [−180, 180], representing the difference between the current estimated heading and the desired heading toward the goal. To perform the task, we used a convolutional neural network (CNN, Supplementary Table 1), with the custom VGG 19 architecture^47^ in which we replaced the Softmax classifier with a regression classifier because our output was an angle between [−180 – 180]. We used a ‘transfer learning’ approach, meaning that the first convolutional layers were already pre-trained^48^. We used a Relu activation function, and L2 regularization penalty, with a learning rate of 0.01 and a batch size of 32 for training.

The loss function was modeled as:

1 $${{{{{\rm{loss}}}}}}=\mathop{\sum }\limits_{{{{{{\rm{image}}}}}}\,0}^{{{{{{\rm{image}}}}}}\,31}\left\{\frac{{({{{{{\rm{ang}}}}}}\,{{{{{\rm{diff}}}}}})}^{2}}{2 * 32}+1{{{{{{\rm{e}}}}}}}^{-4}\cdot ({{{{{\rm{regulariztion}}}}}}\,{{{{{\rm{losses}}}}}})\right\}$$

where: ang diff was defined as ${{\min }}\left\{\left|{angel}1-{angel}2\right|,360-\,\left|{angel}1-{angel}2\right|\right\}$ and angel1, angel2 are the correct label and the output of the network accordingly.

### Estimating the distributions of artificial neurons’ activation

To assess the directional response of the unit, we fed our network with images from all locations and measured the average response of each neuron from all 360° angles relative to the target (averaged across all locations). To identify directional units, we binned and smoothed the activation with bins of 30 degrees and used the Rayleigh test for non-uniformity of circular data. We then used a peak detection function (Matlab) to classify the directional neurons to unimodal, bimodal, and multimodal neurons according to the number of peaks that were detected using the following criteria: a minimum distance between peaks of 20°, a minimum peak height of 0.05, and a minimum peak prominence of 0.065 (peak values were normalized to 0–1). The width of the neuron was measured at half of the height of the peak (on both of its sides).

To plot the average activation pattern, we examined the distribution of the azimuth-dependent activation patterns using a bin of 20 degrees (Fig. 3b) and then only used neurons in the bin with the highest probability to show the average of the azimuth-dependent activation patterns (Fig. 3c).

### The bat-simulator

The simulator was run as described in the main text. However, because the database did not contain data from all possible headings in all the locations along the path, when the exact image was not available, we took the nearest location in both location and azimuth. As the simulated bat never moved far from the original trajectory, this was a good approximation.

### Statistics and reproducibility

To obtain visual information we flew the drone along 1 km track at seven locations along the last 11 km of the bat’s route. Every 250 m we stopped and rotated the drone 360° to acquire images from more angles. A year later we collected additional data from 9 points that were located farther away from the bat’s trajectory (2–5 km) to test generalization in both space and time. Neural network analysis was conducted using TensorFlow platform in Python. All other statistical analyses were conducted using Matlab 2019b. Detailed information regarding all statistical tests are noted in the text.

### Reporting summary

Further information on research design is available in the Nature Portfolio Reporting Summary linked to this article.

## Supplementary information

Supplementary Information

Reporting Summary

## Data Availability

All dataset and code generated during the current study are available in the Mendeley repository, 10.17632/5wc4vphw7j.1.
