# Supplementary material for: An artificial neural network explains how bats might use vision for navigation
Source: Commun Biol. 2022 Dec 3;5:1325. doi: 10.1038/s42003-022-04260-5 (PMC9719490; doi:10.1038/s42003-022-04260-5)
Supplement: Supplementary file 1 — Supplementary Information [file 42003_2022_4260_MOESM1_ESM.pdf]

Supplementary Information for

An artificial neural network explains how bats might use vision for navigation

Aya Goldshtein[14], Shimon Akrish[2], Raja Giryes[2] & Yossi Yovel[1,3*]

[1] School of Zoology, Faculty of Life Sciences, Tel Aviv University, Tel Aviv 6997801, Israel
[2] School of Electrical Engineering, Faculty of Life Sciences, Tel Aviv University, Tel Aviv 6997801, Israel
[3] Sagol School of Neuroscience, Tel Aviv University, Tel Aviv 6997801, Israel
[4] Present address: Department of Collective Behavior, Max Planck Institute of Animal Behavior, Konstanz 78464, Germany

* Corresponding author: yossiyovel@gmail.com

This PDF file includes:

Supplementary Figures 1 to 3

Supplementary Table 1

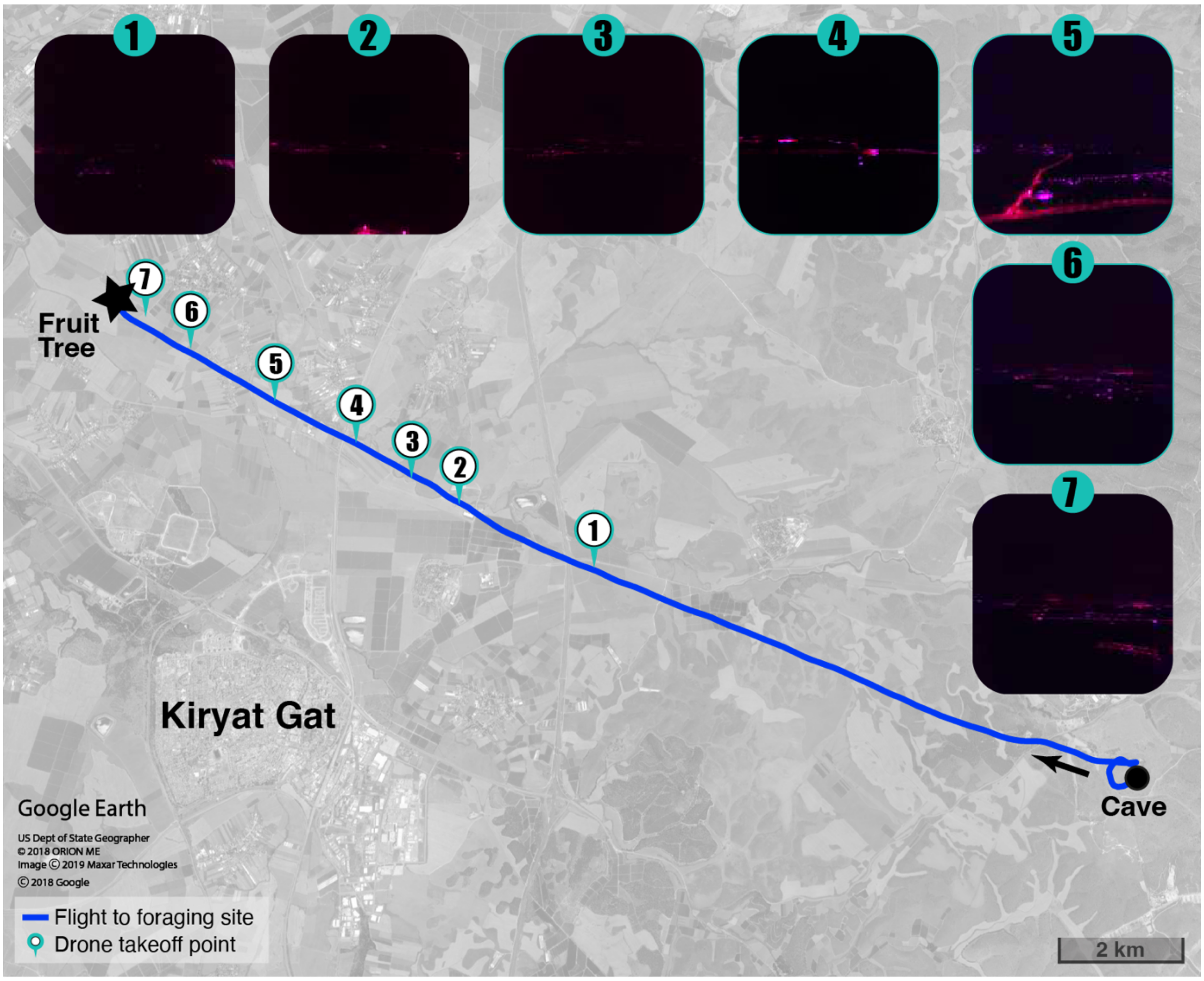

Supplementary Figure 1. Seven visual inputs that were taken from an angle of 30° in relation to the target at different locations throughout the flight. The blue line shows the bat's actual trajectory from its cave (black circle) to its target tree (black star). Seven green points represent the location where the drone took off. Images with green frames show examples of input-images that were taken at 30° relative to the target. Note how different these images are from one another and the network always pointed towards the right direction. The satellite image was obtained from Google Earth, 2018.

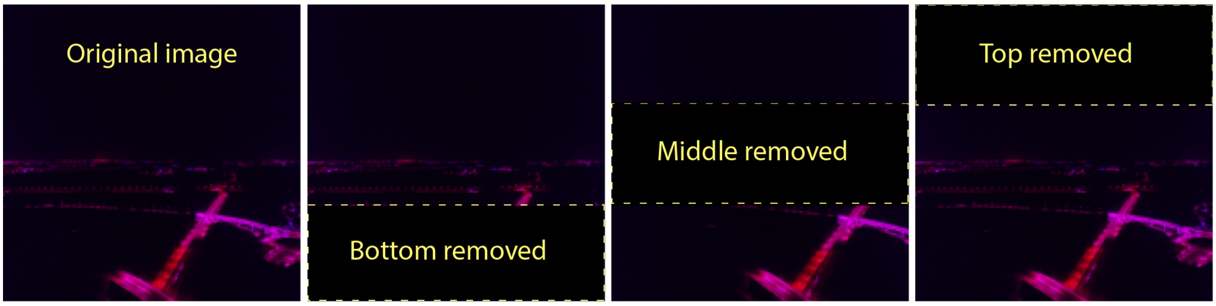

Supplementary Figure 2. Examine which part of the images was most important for navigation. Examination of different parts of the image (bottom, middle, top) provided insight regarding which part was most important for the navigation-net.

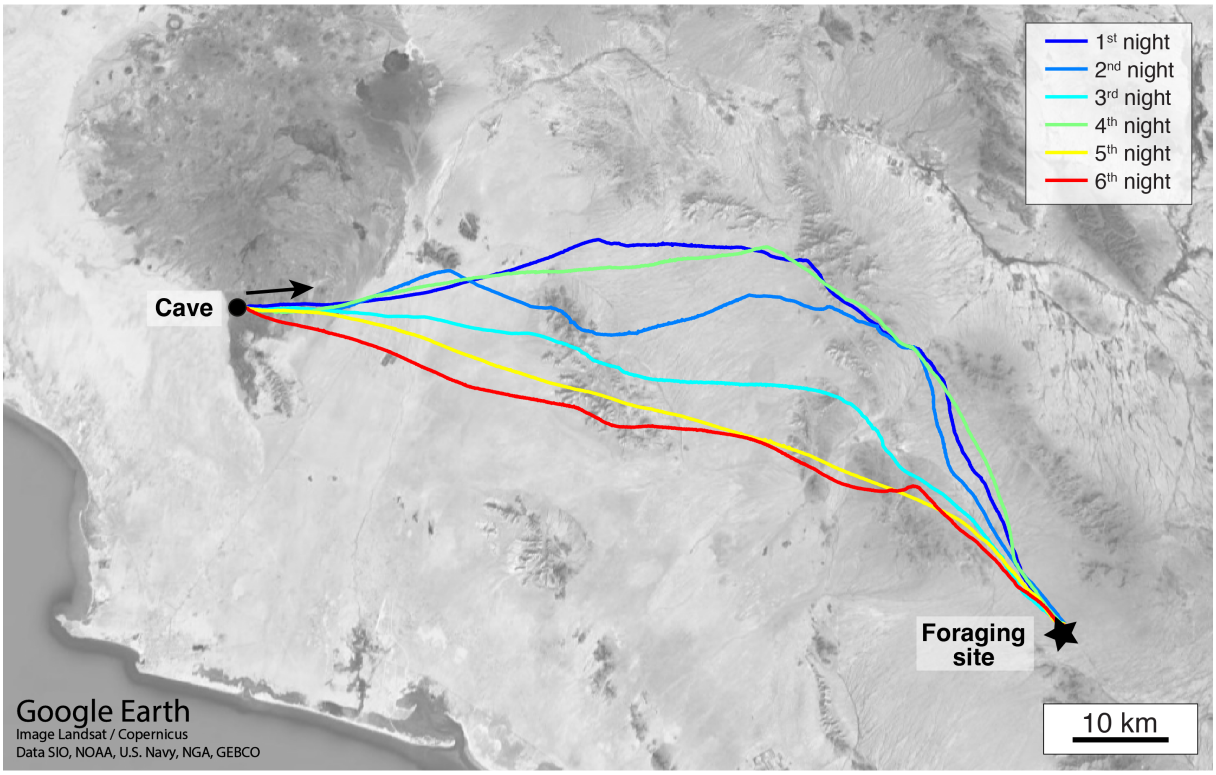

Supplementary Figure 3. Six nights of a Lesser long-nosed bat (*Leptonycteris yerbabuenae*) flying from its roost to a foraging site. Colors depict different nights. We tracked this bat as it switched sites and thus probably returned to this site for the first time this season after not visiting it for a year. Note how the trajectory becomes more direct during the 5th and 6th nights. The satellite image was obtained from Google Earth.

Layer	Pre-trained
Conv 3-64<br>Conv 3-64	Yes
MaxPool 1 + Dropout	
Conv 3-128<br>Conv 3-128	Yes
MaxPool 2 + Dropout	
Conv 3-256<br>Conv 3-256<br>Conv 3-256<br>Conv 3-256	Yes
MaxPool 3 + Dropout	
Conv 3-512<br>Conv 3-512<br>Conv 3-512<br>Conv 3-512	Yes
MaxPool 4 + Dropout	
Conv 3-512<br>Conv 3-512<br>Conv 3-512<br>Conv 3-512	Yes
Conv 3-512<br>Conv 3-512	No
MaxPool 5 + Dropout	
FC-4096	No
Dropout	
FC-1024	No
Dropout	
FC-256	No
Dropout	
FC-1	No

Supplementary Table 1. The architecture of the neural network

FC = fully connected. Conv = convolution.
