# Supplementary material for: An artificial neural network explains how bats might use vision for navigation
Source: Commun Biol. 2022 Dec 3;5:1325. doi: 10.1038/s42003-022-04260-5 (PMC9719490; doi:10.1038/s42003-022-04260-5)
Supplement: Supplementary file 2 — Reporting Summary [file 42003_2022_4260_MOESM2_ESM.pdf]

Corresponding author(s): Yossi Yovel

Last updated by author(s): Nov 2, 2022

Reporting Summary

Nature Portfolio wishes to improve the reproducibility of the work that we publish. This form provides structure for consistency and transparency in reporting. For further information on Nature Portfolio policies, see our Editorial Policies and the Editorial Policy Checklist.

Statistics

For all statistical analyses, confirm that the following items are present in the figure legend, table legend, main text, or Methods section.

n/a	Confirmed	
☐	☒	The exact sample size (*n*) for each experimental group/condition, given as a discrete number and unit of measurement
☐	☒	A statement on whether measurements were taken from distinct samples or whether the same sample was measured repeatedly
☐	☒	The statistical test(s) used AND whether they are one- or two-sided<br>*Only common tests should be described solely by name; describe more complex techniques in the Methods section.*
☐	☒	A description of all covariates tested
☐	☒	A description of any assumptions or corrections, such as tests of normality and adjustment for multiple comparisons
☐	☒	A full description of the statistical parameters including central tendency (e.g. means) or other basic estimates (e.g. regression coefficient) AND variation (e.g. standard deviation) or associated estimates of uncertainty (e.g. confidence intervals)
☐	☒	For null hypothesis testing, the test statistic (e.g. F, t, r) with confidence intervals, effect sizes, degrees of freedom and P value noted<br>*Give P values as exact values whenever suitable.*
☒	☐	For Bayesian analysis, information on the choice of priors and Markov chain Monte Carlo settings
☒	☐	For hierarchical and complex designs, identification of the appropriate level for tests and full reporting of outcomes
☒	☐	Estimates of effect sizes (e.g. Cohen's *d*, Pearson's *r*), indicating how they were calculated

Our web collection on statistics for biologists contains articles on many of the points above.

Software and code

Policy information about availability of computer code

Data collection	We did not used code for data collection
Data analysis	Neural network analysis was conducted using TensorFlow platform in Python. All other statistical analyses were conducted using Matlab 2019b.

For manuscripts utilizing custom algorithms or software that are central to the research but not yet described in published literature, software must be made available to editors and reviewers. We strongly encourage code deposition in a community repository (e.g. GitHub). See the Nature Portfolio guidelines for submitting code & software for further information.

Data

Policy information about availability of data

All manuscripts must include a data availability statement. This statement should provide the following information, where applicable:

- Accession codes, unique identifiers, or web links for publicly available datasets
- A description of any restrictions on data availability
- For clinical datasets or third party data, please ensure that the statement adheres to our policy

The dataset generated and analyzed during the current study is available in the Mendeley repository, DOI: 10.17632/5wc4vphw7j.1.

Field-specific reporting

Please select the one below that is the best fit for your research. If you are not sure, read the appropriate sections before making your selection.

☒ Life sciences ☐ Behavioural & social sciences ☐ Ecological, evolutionary & environmental sciences

For a reference copy of the document with all sections, see nature.com/documents/nr-reporting-summary-flat.pdf

Life sciences study design

All studies must disclose on these points even when the disclosure is negative.

Sample size	To obtained visual information we flew the drone along 1km track at seven locations along the last 11 km of the bat's route. Every 250 m we stopped and rotated the drone 360° to acquire images from more angles. A year later we collected additional data from 9 points that were located farther away from the bat's trajectory (2-5 km) to test generalization in both space and time.
Data exclusions	NA
Replication	Visual data was collected during two years and data was compared across these years
Randomization	NA
Blinding	Data collection was conducted blindly regarding the expected light conditions and the expected images that were recorded at each location and in line with flight regulations in the area.

Reporting for specific materials, systems and methods

We require information from authors about some types of materials, experimental systems and methods used in many studies. Here, indicate whether each material, system or method listed is relevant to your study. If you are not sure if a list item applies to your research, read the appropriate section before selecting a response.

Materials & experimental systems

n/a	Involved in the study	
☒	☐	Antibodies
☒	☐	Eukaryotic cell lines
☒	☐	Palaeontology and archaeology
☐	☒	Animals and other organisms
☒	☐	Human research participants
☒	☐	Clinical data
☒	☐	Dual use research of concern

Methods

n/a	Involved in the study	
☒	☐	ChIP-seq
☒	☐	Flow cytometry
☒	☐	MRI-based neuroimaging

Animals and other organisms

Policy information about studies involving animals; ARRIVE guidelines recommended for reporting animal research

Laboratory animals	NA
Wild animals	We used GPS trajectory of one adult male Egyptian Fruit bat (Rousettus aegyptiacus). The bat was caught when emerging from its cave using a mist net, a GPS device was attached to its back using surgical cement. A telemetry unit was attached to the GPS to assure its recovery after falling off the bat.
Field-collected samples	NA
Ethics oversight	The study was performed according to the permit of the Tel-Aviv University IACUC (Number: L-11-054).

Note that full information on the approval of the study protocol must also be provided in the manuscript.
